# User experiences of a mobile phone-based health information and surveillance system (mHISS): A case of caregivers of children under-five in rural communities in Ghana

**DOI:** 10.1371/journal.pone.0261806

**Published:** 2022-01-21

**Authors:** Emmanuel Acquah-Gyan, Princess Ruhama Acheampong, Aliyu Mohammed, Timothy Kwabena Adjei, Emmanuel Agyapong, Sampson Twumasi-Ankrah, Augustina Sylverken, Michael Owusu, Ellis Owusu-Dabo

**Affiliations:** 1 Department of Global and International Health, College of Health Sciences, School of Public Health, Kwame Nkrumah University of Science and Technology, Kumasi, Ghana; 2 Department of Epidemiology and Biostatistics, College of Health Sciences, School of Public Health, Kwame Nkrumah University of Science and Technology, Kumasi, Ghana; 3 Kumasi Centre for Collaborative Research in Tropical Medicine (KCCR), Kumasi, Ghana; 4 Department of Statistics and Actuarial Science, College of Science, Kwame Nkrumah University of Science and Technology, Kumasi, Ghana; 5 Department of Theoretical and Applied Biology, College of Science, Kwame Nkrumah University of Science and Technology, Kumasi, Ghana; 6 Department of Medical Diagnostics, College of Health Sciences, Kwame Nkrumah University of Science and Technology, Kumasi, Ghana; Unaizah College of Pharmacy, Qassim University, SAUDI ARABIA

## Abstract

**Background:**

The rapid advancement of mobile technology has fueled the use of mobile devices for health interventions and for improving healthcare provision in underserved communities. Despite the potential of mHealth being used as a health information and surveillance tool, its scale-up has been challenging and, in most cases, unable to advance beyond the pilot stage of implementation. The purpose of this study was to explore user experiences of a mobile phone-based interactive voice response (IVR) system among caregivers of children under-five in rural communities in the Asante Akim North District of Ghana.

**Methods:**

The study adopted an exploratory design. A convenience sampling technique was used to recruit 35 participants who had used an IVR system for at least six months for the study. About 11 in-depth interviews and three focus group discussions were conducted among participants using a semi-structured interview guide. Thematic content analysis was utilized for the analysis of data in this study.

**Result:**

The system was found to be acceptable, and the attitude of caregivers towards the system was also positive. The study discovered that the mobile phone-based Health Information and Surveillance System (mHISS) was useful for improving access to healthcare, communicating with health professionals, served as a decision support system, and improved caregivers’ awareness about self-management of childhood illnesses. Poor network quality, unstable electricity power supply, and dropped/cut calls served as significant barriers to using the mHISS system.

**Conclusion:**

The mHISS system was generally acceptable and could help improve access to healthcare and identify children with severe health conditions during outbreaks of diseases.

## Background

Even though there has been a substantial global reduction in childhood mortalities over the past decade, it remains a major public health problem for many countries, especially sub-Saharan African countries [1, 2]. In 2018, under-five mortality contributed to about 5.3 million deaths globally, with the majority occurring in sub-Saharan Africa, mostly to preventable infectious diseases [1, 2].

In sub-Saharan African countries, including Ghana, access to health care for patients and caregivers is very challenging. The health care delivery systems are confronted with numerous challenges that create limited universal access to healthcare and weaker surveillance systems resulting in delays to respond to childhood diseases. Health staff shortages, long travel distances to health facilities, and the high cost of health care, among others, are known barriers to accessing healthcare in sub-Saharan Africa [3, 4]. These challenges prevented sub-Saharan African countries from achieving the millennium development goal four (MDG 4); to reduce child mortality rate by two-thirds between 1990 and 2015 [5]. Therefore, to contribute to achieving the sustainable development goal three, target two (SDG 3.2); reduce under-five mortality to at least as low as 25 deaths per 1000 live births by 2030, innovative interventions are required to curb the persisting health care challenges and support universal access to healthcare, proper surveillance, and timely diagnosis and treatment of diseases of children under-five [3, 4, 6]. The rapid advancement of mobile technology has fueled the use of mobile devices for health interventions and for improving healthcare provision in underserved communities [7–9]. MHealth; the use of mobile technology in healthcare, has been one of the innovative interventions that have proven to be useful in the areas of promoting health care delivery, health care accessibility, and health equity among vulnerable and underserved populations [9–11]. However, sustaining, up-scaling, and integrating mHealth interventions into broader health care systems have been a major challenge in many developing countries. The majority of the interventions are not able to progress beyond the pilot stage of implementation and often cover a small-targeted scope [11–13]. Primarily, in most sub-Saharan African countries, they are implemented without an understanding of user experiences regarding its acceptance, usefulness, and challenges among its end users [14–17]. This has made it difficult for health systems in developing countries to realize the full benefits of mHealth being used as a health information and surveillance system.

This is a nested study designed with the main purpose to explore user experiences of a mobile phone-based interactive voice response (IVR) system with regards to user acceptance and attitudes, usefulness, and associated challenges among caregivers of children under-five in the Asante Akim North District (AAND) of Ghana.

### The mobile phone-based health information and surveillance system (mHISS)

The mHISS is implemented in a bigger prospective study titled; *"Reducing child mortality*: *the role of mobile electronic health information system (MHIS)”* (MobChild Project) in the AAND of Ghana. The "*Mobchild project*" is a multi-dimensional quasi-experimental study that utilizes the packages of weekly health education voice messages and a toll-free IVR call system to improve under-five survival in AAND and inform strategies for national uptake. The health education voice messages are aimed at helping build caregiver capacities in identifying childhood disease danger signs, while the IVR system serves as a mHISS for the district health authorities (DHAs) to have access to real-time data on childhood diseases for planning interventions and monitoring disease surveillance.

The mHISS/IVR technology is designed based on a clinical algorithm using the decision trees of Integrated Management of Childhood Illnesses (IMCI) [18] as guidelines. The system allows caregivers to call into a computer system to report disease symptoms of their children through voice prompts provided by the system and respond to them using a mobile phone keypad for a health advice [19]. Users have the opportunity to select a language of their choice; Twi (a local language) or English for interacting with the system. They then respond to several sequential questions on childhood disease symptoms through the IVR system. The system captures common childhood disease symptoms such as fever, cough, diarrhea, vomiting, unconscious or convulsions, etc.

The system then classifies reported disease symptoms as severe, mild, or moderate and therefore provides advice to the caregiver whether to seek immediate healthcare or provide home care and seek proper care only when symptoms persist. The system’s advice is dependent on the classification of the severity of symptoms reported [19]. For example, disease symptoms such as inability to breastfeed or drink, unconsciousness, or convulsions are classified as danger signs by the system and caregivers are then advised to immediately take their child(ren) to the nearest health facility. Another symptom like fever less than three days is advised to provide home care treatment such as sponging and paracetamol administration and seek proper care if symptoms persist after 24 hours.

After the advice by the system, caregivers are further given the option to end the interaction with the system or press a particular keypad to link the interaction to a Pediatrician for further interaction and guidance on symptoms reported. The system keeps records of all reported disease symptoms on a dashboard for access by the DHAs. The DHAs use the real-time data collected from caregivers in the system for planning and management to improve childhood disease outcomes.

In this study, the researchers only explored caregivers’ experiences concerning the IVR system implemented by the "*Mobchild project*".

## Methods

### Study setting

This study was carried out in the AAND in the Ashanti Region of Ghana. The AAND was established in 2012 as one of the operating Districts in the Ashanti Region. It lies between latitudes ‘60 30’ and ‘70 30’ North and longitudes ‘00 15’ and ‘10 20’ West. It covers a land area of 1,126 square kilometers [20]. The District has a projected population of about 87866 and 115 communities. About 53.5 percent of dwellings for the population are rural. Communities such as Agogo, Juansa, Domeabra, Akutuase, Wioso, Hwidiem etc., are among the most easily accessible communities in the District. The towns are ruled by traditional Chiefs and Queen mothers [20].

The Akan tribe is the most common ethnic group in the District. Other minority ethnic groups include Fantis, Ewes, Gas, Kussasis, Dagombas, etc. The predominant local language for communication in the District is Twi. Other languages such as Ewe, Nzema, Dagomba etc., are also used for communication in the District. The population in the District are predominantly farmers and have considerable access to water, electricity and mobile cellular network. The healthcare delivery system of the District boast of 12 health facilities; one missionary Hospital, four Health Centers and seven Community-based Health Planning and Services (CHPS) [20].

### Population, inclusion and exclusion criteria

The population of this study consisted of caregivers of children under-five; individuals performing parenting responsibilities for children under-five in AAND of Ghana recruited on the *“MobChild project”*.

Participants for this study consisted of caregivers of children under-five who had utilized the IVR system implemented by the *“MobChild project”* at least once after the implementation of the system to seek healthcare for their children.

Participants on the *“MobChild project”* who had never used the implemented IVR system and those who had ever utilized the system but were unwilling to be part of the study were excluded.

### Study design and approach

The study was a nested exploratory qualitative study that utilized two qualitative data collection techniques; focus group discussions (FGDs) and in-depth interviews (IDIs), to explore user experiences of caregivers of children under-five regarding the use of an IVR system. The study adopted this design to help derive context-based in-depth understanding and insight into the use of the IVR system implemented in the *“MobChild project”*. The study adopted IDIs as a data collection strategy to cater for the cultural circumstances that restricted some participants with very young children (below two weeks) from moving outside of their home environment to partake in the FGDs. FGDs were also adopted to encourage brainstorming to gather several and adequate caregivers’ experiences concerning the use of the IVR system.

A total of 35 participants recruited using the convenience sampling technique participated in this study. Participants were conveniently selected based on the easy geographical accessibility of their communities and their ready availability for the study. The sample size was informed by the saturation of responses from participants during data collection [21]. Researchers recruited participants for the IDIs and FGDs until no new additional information was being obtained (saturation) from the last two IDIs, and the last FGD.

### Data collection procedures

Telephone numbers of some of the caregivers who met the inclusion criteria were retrieved from the IVR system’s dashboard of the *“MobChild project”*. The researchers made follow-up calls to such caregivers and sought their permission to meet them in their respective communities. After their permission was sought, the researchers met them in their communities and briefed them on this study nested in the *“MobChild project”* on which they are already serving as participants.

Using languages with which target participants were conversant (Twi or English), and the assistance of Witnesses, the researchers explained the aim and objectives of the study and the benefits and cost associated with the study to target participants. Researchers assured them of confidentiality and anonymity and sought their voluntary consent to partake in the study. Caregivers willing to participate in the study were then made to sign or thumbprint two voluntary consent forms: one for researchers and one for participants. Witnesses were also made to sign on the consent forms of the people they witnessed consenting to partake in this study. Caregivers who met the criteria for the IDIs were interviewed in their homes free from third parties while arrangements were made with other caregivers regarding when the FGDs could be organized.

The researchers then secured a conference room conducive for discussions in Agogo Presbyterian Hospital; a local district hospital for the FGDs.

Three experienced research assistants were trained together to assist with data collection and transcription to minimize biases. The study conducted 11 IDIs, and three semi-structured FGDs among a total of 24 caregivers (eight caregivers in a group). The interviews and discussions were completed in Twi and were translated to English for analysis during transcription. They were limited to a maximum of 30 minutes per an IDI and 60 minutes per a FGD. This was to ensure an adequately controlled data collection process and the comfort of participants with very young children. This, however, did not affect the quality of the data collection process in this study. Data collection was conducted from 1^st^– 30^th^ August 2019. The trained research assistants first translated the recorded data. Three postgraduate researchers experienced in qualitative data analyses cross-checked the transcripts thoroughly against their recorded audio files to ensure the accuracy of the transcripts before analysis. This was done to ensure the trustworthiness of the study [22].

The IDIs and FGDs were done using a semi-structured interview guide and recorded using an audiotape recorder. The Unified Theory of Acceptance and use of a Technology (UTAUT) model served as the theoretical framework that guided the design of the interview guide [23]. After a thorough literature search, the UTAUT model emerged as one of the key models with the potential to predict user behavior to a technological system among potential users. Some key questions regarding factors that contribute to user behavior to health technologies propounded in the UTAUT model were qualitatively assessed to predict user experiences in this study. Main questions concerning acceptance and attitude and the benefits and challenges associated with mHealth systems were broadly stated. The broad questions were then narrowed down into simple qualitative questions. The first draft of the interview guide was then developed and shared with local researchers for their inputs on context, feasibility and validity. A pre-test of the data collection instruments was done among six users of the mHISS intervention. These caregivers were not again recruited as participants in this study. Minor errors regarding the rewording of some of the questions were made after the pre-test to suite the cultural context of the study.

### Data analysis procedure

This study adopted a thematic content analysis strategy for analyzing the study data [24]. The researchers independently coded the transcripts together with the field notes from the IDIs and FGDs as a single unit. They then dialoged on the codes developed by each researcher. The coding differences, which implied different understanding among the researchers, were further discussed and recoded until inter-coder reliability was achieved. This also contributed to the reflexivity of the study.

The accepted codes were then categorized and themed by the researchers. The derived themes were further reviewed and evaluated among researchers to ensure no theme was left out in the data. Also, before the derived themes were finally accepted to be analyzed, they were accepted by ten of the study participants as a true reflection of what transpired in the interviews and discussions.

### Ethical considerations

Ethical approval for this study was obtained from the Committee on Human Research Publication and Ethics of the Kwame Nkrumah University of Science and Technology (Ref: CHRPE/AP/562/19). Written consent was obtained from every participant involved in this study.

Anonymity and confidentiality were also adhered to in this study. Participants’ identifiers; names, titles, contacts etc., were not collected and used for analyses. Information obtained from participants were also kept from third parties on a password-protected laptop.

## Results

### Socio-demographic profile of participants

A total of 35 women aged between 15 and 50 years (Mean 29.6years; Standard deviation [SD] ± 8.9) participated in the study. Participants were from six different communities in the District. Majority of participants were married (n = 18; 51.9%), had attained Junior High school level of education (n = 22; 63%) and belonged to the Akan ethnic group (n = 31; 88.9%). Participants were employed in varied occupations, with the majority (n = 21; 59.2%) being Traders. This is presented in Table 1 below.

**Table 1:** Socio-demographic profile of study participants.

Characteristics	Frequency N = 35	Percentage %
**Gender:**		
Females	35	100
**Age:**		
15–25	13	37.1
26–35	13	37.1
36–45	6	17.1
>45	3	8.6
Mean (SD)	29.63 (± 8.99)	
**Marital status:**		
Single	4	11.4
Married	18	51.4
Divorced	1	2.9
Cohabiting	12	34.3
**Level of Education:**		
Primary	4	11.4
JHS/JSS	22	62.9
SHS/SSS	9	25.7
**Occupational status:**		
Unemployed	5	14.3
Students	2	5.7
Trader	21	60
Farmer	6	17.1
Seamstress	1	2.9
**Religion:**		
Christian	34	97.1
Muslim	1	2.9
**Ethnic group:**		
Akan	31	88.6
Kusaasi	4	11.4
**Communities:**		
Agogo	16	45.7
Hwidiem	4	11.4
Juansa	5	14.3
Domeabra	4	11.4
Wioso	3	8.6
Akutuase	3	8.6

### Theme 1: Acceptance of, and attitude towards the mHISS

The majority of participants accepted the system, and their attitude towards it was also positive. These were found based on findings on sub-themes such as willingness to use the system, the ease of use of the system, adherence to the system’s advice, and social influence towards the system. Findings on the sub-themes are stated below:

#### Willingness to use the system

Participants found the system to be a very useful tool and expressed their willingness to continue seeking healthcare for their children through the system. Participants were willing to recommend the system to their relatives and friends and advocate for the system’s availability to all caregivers in Ghana. These were found through the quotes below:

*"Yes*. *We will be glad to recommend the system to others because if someone is dying*, *you can’t let them die"* (40-year-old caregiver, Agogo, FGD).

*"Yes*. *The system is for children below five years*. *So if you have a brother or a sister and they also have a child like that*, *then you can give the number (the toll-free number) to them"* (47-year-old caregiver, Agogo, FGD).

*"Yes*. *I have two relatives; my mother and my sister*, *they have kids like mine*, *and I want to help them register so that they can benefit from the system as well"* (21-year-old caregiver, Hwidiem, IDI).

#### Ease of use of the system

Overall, participants reported the IVR system was easy to use. The majority used the system themselves without any assistance. The inclusion of a local language and clear interaction voices used in the system’s design facilitated the ease of using the system. The quotes below were expressed by some participants when asked how easy it was to use the system:

*"…when you dial the number and it goes through*, *the system will respond and ask you whether you want English or Twi*. *So it’s up to you to choose that you like Twi*. *Once you select Twi*, *it will start asking you questions about the disease of your child in Twi*, *and it is easy to understand"* (35-year-old caregiver, Agogo, FGDs).

*"When I called*, *the system was telling me what to do*. *For example*, *when I called*, *the system told me if your child is vomiting*, *press one and so on*, *which was very clear and easy to me"* (23-year-old caregiver Agogo, IDI).

*"When I called*, *the system responded immediately*. *Everything with the system was clear to me*, *and there were no challenges"* (21-year-old caregiver, Agogo, IDI).

#### Adherence to the system’s advice

A high adherence to the advice given by the mHealth system among the majority of participants showed acceptance and a positive attitude towards the system. The majority of participants advised by the system abided by the instructions given by the system.

*"…for me*, *when I called and was asked to take my child to the hospital*, *I did so”* (26-year-old caregiver, Juansa, FGD).

*"When I called*, *the Doctor asked me to give him oral rehydration solution (ORS) and wait till the morning to take him to the hospital if the situation persists*. *So I gave him the ORS*, *and by the morning*, *he was OK"*. (28-year-old caregiver, Agogo, FGD).

*"… so when I called*, *the Doctor asked me to take him to the hospital*. *I took him to the hospital*, *they gave her an injection*, *and all the rashes went away*.*"* (20-year-old caregiver, Hwidiem, FGD).

#### Social influence (influence by significant others)

The social influence on the system was found to be positive. The system had received approval from significant others of participants and was also perceived as a useful tool for seeking healthcare. Close relatives of participants who were not part of the bigger study perceived the IVR system positively, and beneficial for accessing healthcare. Therefore, participants were encouraged to continue using the system. The quotes below support this:

*"On the first day that I called the system and was asked to rush my child to the hospital*, *my sister started expressing her desire and wished that she had also been registered*. *All the others around me were all happy"* (21-year-old caregiver, Hwidiem, FGD).

*“It was even my husband who reminded me of the number to call the system and asked me to call to speak with the Doctor when my child fell sick*. *So he is happy with the system*. *He also sees the system as beneficial"* (25-year-old caregiver, Agogo, FGD).

*"…my husband is also happy with the system*. *In the night when my child wasn’t feeling well*, *it was him who called the system with his phone before giving it to me to speak with the Doctor"* (42-year-old caregiver, Juansa, FGD).

### Theme 2: Usefulness of the mHISS to its users

Almost all participants expressed how useful the system was to them. The benefits derived from the system according to participants have been grouped under sub-themes below:

#### Access to healthcare/improved health

The system was found to have helped most caregivers seek and provide healthcare for their sick children during periods that access to a health facility was difficult. This had helped them to manage the health conditions of their children and prevented it from worsening.

*"It was helpful to me*. *It was in the night when my child had a fever and diarrhea*. *So I called*, *and the Doctor told me if I have ORS and paracetamol syrup*, *I should give the child some to drink*. *I gave them to her*, *and the fever went down"*. (23-year-old caregiver, Agogo, FGD).

*"For me*, *when I called*, *the Doctor asked me to give him ORS and wait until the morning to take him to the hospital if the situation persists*. *So I gave him the ORS*, *and by the morning*, *he was OK*.*"* (28-year-old caregiver, Agogo, FGD).

*"For benefits*, *I got some from the system*. *This is because when I called the system and did exactly as the Doctor asked me to do for the child*, *he became better*, *and the condition has not occurred again"*. (35-year-old caregiver, Akutuase, FGD).

#### Communicating with a health professional

Almost all participants were able to communicate with a Pediatrician from their homes through the system to seek healthcare for their sick children.

*"When I called the system*, *I was directed to speak to a Doctor*. *I explained the child’s condition to the Doctor*, *and the Doctor also asked me questions concerning my child’s condition"* (18-year-old caregiver, Wioso, IDI).

*"When I called*, *it went through*, *and I was able to communicate with the Doctor*. *He asked me questions about the conditions of my child and then asked me to go to the hospital tomorrow"* (29-year-old caregiver, Agogo, IDI).

#### Decision-making process

Almost all participants reported that the system supported them in making decisions regarding whether their child(ren)’s health condition is severe or not and whether taking the child to a health facility is necessary. They also reported that the system helped them in providing first aid to help care for their children.

*"The system helped me because I first thought my child’s sickness was nothing severe*. *However*, *when I spoke with the Doctor*, *he explained everything to me and asked me to take the child to the nearest hospital or else the condition will worsen"* (23-year-old caregiver, Agogo, FGD).

*"We called the Doctor (System)*, *and he advised us to take the child to the nearest health facility immediately*. *We quickly did that*, *and at the first Health Center*, *they told us my child’s condition was beyond their capacity*. *So they referred us to Agogo Hospital (A district hospital)*. *That was when I realized the child could have died if we had decided to stay home and not have called the Doctor (system)"* (20-year-old caregiver, Hwidiem, IDI).

#### Educating caregivers on the management of childhood disease symptoms

Participants reported that the system taught them about the management of certain common childhood disease symptoms. These were expressed through the quotes below:

*"The system has helped me*. *Like I was saying*, *when my child was vomiting*, *it was the system that taught me to give him ORS*. *If not it*, *I wouldn’t have known what to do"*. (18-year-old caregiver, Agogo, IDI).

*"If you are told to do something that you do not know which involves the life of your child*, *I think it’s very useful*. *The system is very useful because it educated me on how to take care of my child"* (20-year-old caregiver, Agogo, IDI).

#### Emotional support to caregivers

The system was found to have relieved some participants of fear and anxiety. It made them feel relaxed and supported when their children were sick due to the opportunity to communicate with a Doctor.

*"…when my child was refusing to drink the breast milk*, *I was scared*. *So when I called Doctor*, *and he spoke with me*, *he told me not to be scared but take him to the hospital*. *That made me feel OK"*. (29-year-old caregiver, Agogo, FGD).

*"…this is my firstborn*. *So when he was suddenly vomiting and having diarrhea in the night*, *I was terrified*. *However*, *when I called and got the chance to talk to the Doctor*, *he calmed me down and asked me to prepare ORS for him to drink*. *So I felt OK and did exactly what he told me to do”*. (25-year-old caregiver, Agogo, FGD).

### Theme 3: Barriers to the use of the mHISS

In this study, the perceived and actual barriers reported by participants regarding the system have been sub-themed below:

#### Poor infrastructure

The majority of participants reported that a lack of stable and quality cellular network and a reliable electricity supply made utilizing the system when their children were sick challenging. Participants expressed this through the quotes below:

*"The only thing is*, *when it rains heavily*, *we have network problems*. *This made it difficult to call the system…"* (35-year-old caregiver, Akutuase, IDI).

*"The network can be bad sometimes*. *However*, *it does not take long to stabilize*, *so that may not be a big challenge*. *But with light (Electricity) issues*, *it is a big problem"* (48-year-old caregiver, Domeabra, IDI).

*"The network is bad here*, *so it was very difficult for me to call"* (18-year-old caregiver, Wioso, IDI).

#### Dropped calls

To enjoy the full benefits of IVR systems, the need for sustained interaction with the system is relevant. Therefore, in a situation where a user cannot interact with the system due to dropped calls, then the full benefit of the system is curtailed. Some Participants expressed the quotes below:

*"When we called*, *we were on the line but didn’t hear anything*, *and then the call dropped*. *Meanwhile*, *my sister’s child was also seriously sick…” (*40-year-old caregiver, Agogo, IDI).

*"At times*, *the child may be severely sick*. *So when we call and the line drops*, *then it becomes a great challenge"* (42-year-old caregiver, Juansa, IDI).

#### Limited system understanding and difficulty pressing keys

Some participants reported having challenges with understanding the system and pressing phone keypads to feed information into the system.

*"I did not understand the system from the initial stage*. *I didn’t even know what to do if to press 1 or 2"* (20-year-old caregiver, Hwidiem, IDI).

*"I was told to press 1 or 2 when I called the system*. *Pressing those numbers also disconnected the line*. *My only problem is the pressing of the numbers"*. (48-year-old caregiver, Domeabra, IDI).

#### Unmet expectations

Some participants expressed their worry about the inability of the IVR system to cater for other disease symptoms, e.g. symptoms of eye infections, and prescribe a specific brand of drugs. Therefore, it became challenging to some participants when this expectation was not met.

*"There was no information on the eye condition of my child*, *so I couldn’t press any key on the situation"* (18-year-old caregiver, Wioso, IDI).

*"To me*, *I was expecting the system to prescribe a particular drug for my child’s condition*, *but it was not so"*. (35-year-old caregiver, Akutuase, IDI).

#### Delays by the system during emergencies

Almost all participants expressed the challenge of having to go through lengthy procedures of the IVR system by pressing numerous keys before getting the opportunity to speak to a Doctor. They expressed this may be a challenge during times of emergencies.

*"…when the child’s condition is severe and you have to go through all those procedures before the Doctors speak to you*, *then it will be a big problem"* (Caregivers, FGDs & IDIs).

## Discussion

The main objective of this study was to explore user experiences of a mobile phone-based IVR system with regards to acceptance, usefulness, and associated challenges. To the best of our knowledge, this is one of the first studies that qualitatively explored user experiences of an IVR system with human to human interaction incorporated into it. Previous studies focused on feasibility and usability and user experiences of systems without a human to human interaction incorporation [16, 17, 19].

Prior to this study, mobile phone subscriptions had been reported to be high globally [25]. In Ghana, the same had also been reported [26]. This, however, creates opportunities for mHealth interventions to be designed and implemented globally to harness the challenges of health systems, especially in developing countries.

The study found the mHISS system acceptable, and users expressed a positive attitude towards it. The system increased access to healthcare for caregivers of children under-five and promoted communication between caregivers and physicians. The system was also found to have supported caregivers in making healthcare decisions concerning their children. The mHISS system serving as a tool to have improved awareness of caregivers on the management of childhood disease symptoms, and providing emotional support to caregivers was also found. In literature, other studies have reported similar benefits that mHealth interventions targeted at less-resourced settings are useful in enhancing access to healthcare, improving caregiver awareness on the management of childhood disease symptoms, providing emotional support and assisting caregivers in making healthcare decisions [11, 16, 17, 27].

The usefulness of the IVR system evaluated in this study makes it an innovative and efficient tool that could improve the health of populations with limited health resources. The system could reduce healthcare access burdens such as time and transport costs among caregivers and improve the health of children under-five even during healthcare provider shortages. It could also help caregivers to become active partners in caring for their children. In literature, several studies have also demonstrated the efficiency, feasibility and potential of mHealth systems to reduce healthcare access burdens and improve the health of less-resourced populations [8–10, 28–32], and help caregivers become active partners in caring for their children [32].

The ability of caregivers to manage the disease symptoms experienced by their children was also improved through the system. The system educated caregivers about the severity of disease symptoms of their children and recommended home care treatments for managing minor childhood diseases. It again educated caregivers on the need and urgency to seek proper healthcare for their sick children. This is an indication of the system’s potential to improve the healthcare-seeking behavior of caregivers. Caregivers can act promptly to the disease symptoms of their children without the need to wait for a disease condition to worsen. In situations where a health facility is hard to reach due to either geographical barriers or transport limitations, the mHealth system could support caregivers by recommending healthcare practices to safeguard their children’s health. This reiterates a claim by United Nations women that access to child healthcare services, including first aid and the health education of caregivers, are relevant factors contributing to the attainment of the sustainable development goal three to ensure healthy lives and promote well-being for all at all ages [33].

Among early and inexperienced caregivers who may be anxious and not know what to do when their children are sick, this system could support such caregivers in making decisions and help relieve them from fear and anxiety. The high adherence rate to the system’s advice and the willingness of caregivers to seek healthcare for their sick children indicate how the system could help achieve the above purpose. In sub-Saharan African countries, much of the responsibility for childcare is left to women, especially those who are poor and vulnerable [34]. The mHealth (IVR) system could empower women and enhance their decision-making power to seek healthcare for their children in their households.

The poor state of mHealth infrastructure was discovered as the main barrier to using the IVR system in this study. Using mHealth systems such as IVR systems requires supporting infrastructures such as quality mobile cellular networks and a stable electric power supply. However, in rural and peri-urban communities in Ghana and many sub-Saharan African countries, these facilitating infrastructure have been poorly developed and less invested in [17, 19]. This creates the problems of poor mobile cellular networks and a lack of stable electricity supply to support mHealth initiatives. Therefore, users of mHealth systems become restricted and sometimes demotivated to adopt and use such systems. These barriers could be significant challenges to the sustenance and scale-up of mHealth initiatives in communities. Unsurprisingly, dropped/cut calls were reported by participants as a challenge to their use of the system. This may have been caused by the poor-quality nature of mobile cellular networks reported in the study. In studies conducted in the same setting as this study using a similar IVR system, barriers such as poor mHealth infrastructure and dropped calls were also reported [16, 17, 19]. Another study by Laar *et al*., in a less resource setting in the rural Upper West Region of Ghana, also found the poor state of mHealth infrastructure as a barrier to the effective use of their mHealth system [27].

Familiarity with how mHISS functions and the ability to input information into the system is the backbone of using mobile phone-based IVR systems. The feedback given by a mobile phone-based IVR system to a user depends on the information fed into the system. Therefore, the study finding limited understanding of the system, and difficulty pressing keys to input information as a barrier to some participants makes it worrying. These barriers may have also contributed to the cut/dropped calls, which some participants reported. Mhealth interventional studies implemented in similar settings have also reported limited understanding and familiarity with mHealth systems among end-users [16, 17, 35]. In other studies by Stanton *et al*., and Perosky *et al*., some participants had challenges with pressing buttons, reading keypads of phones, and selecting numbers using functional keys when utilizing an mHealth system [36, 37]. In this study which found the majority (63%) of participants attaining a low level of education (JHS), the issue of literacy cannot be overlooked when limited understanding and difficulty pressing keys are being discussed. In studies by Perosky *et al*., and Crawford *et al*., similar findings regarding the difficulty in understanding and pressing keys to utilize mHealth systems were also reported [37, 38]. In these studies, no or limited literacy among participants again served as a barrier to participants understanding and use of the mHealth systems evaluated.

Unmet expectations and delays by the system during emergencies affected some caregivers’ use of the mHealth system in this study. The mHISS system is not designed to capture all childhood disease symptoms. Therefore, when caregivers utilize the system and the disease symptoms of their children are not listed, this influences their continued use of the system. The unmet expectation of some participants that the mHealth (IVR) system would recommend a specific brand of drugs to them affected some caregivers’ perception and acceptance of the system. A study by Brinkel *et al*., reported a similar finding [17]. In this study, almost half of the participants reported the limitation of their IVR system to address symptoms of other diseases and wished for expansion. The issue of designing a system with the capacity to recommend a specific brand of drugs to caregivers was also reported [17].

Using mobile phone-based IVR systems requires careful interaction by following voice prompt outputs and pressing keypads to input information. This careful process that caregivers would have to complete before receiving health advice from the system and the health professional served as a barrier that could influence the acceptance and use of the system. During childhood disease emergencies, there may not be adequate time for a caregiver to carefully follow all the system’s processes before receiving advice from the system. Therefore, a system that creates rooms for emergency disease situations is relevant to ensure mobile phone-based IVR systems’ effective use, sustainability, and scalability.

Despite all the almost prohibitive barriers confronted by participants in this study, the magnification of the system’s potential benefits may have well-motivated participants to overlook these barriers and to seek healthcare for their children through the system. However, to ensure effective use, sustainability and scalability of the mHealth system, private-public partnerships between governments and telecommunication companies are very relevant. This will ensure the provision and enhancement of needed mHealth infrastructure; quality network and reliable electric power supply to support the sustained use and scale-up of mHealth systems. The active engagement of local community institutions such as families, churches and local health facilities, community health volunteers, and opinion leaders in training end-users of mHealth systems is also significant. The active involvement of local institutions in training end-users of the system will enhance knowledge and understanding regarding the effective use of the system and enhance trust and motivation in the system among users.

### Limitations of the study

The assessment in this study is limited to a mobile phone-based IVR system implemented in rural communities in the Asante Akim North District of Ghana. Therefore, findings from this study cannot be generalized to reflect all mHealth initiatives in the District and Ghana due to the qualitative nature of our research. The convenience sampling technique used, and the recall of experiences by participants regarding the use of the system also exposed our study to selection and recall biases, respectively.

The human-human connection at the end of using the IVR system may have also limited this study. The IVR system may have been found acceptable among participants due to the human-human connection incorporated into it. This, therefore, creates un-answered questions such as;

1. Will the acceptance and attitude towards the system still be the same as discovered in this study or otherwise if the IVR system evaluated in this study was designed without human-human connection, or designed such that users could skip the chatbot and interact directory with the Pediatrician?

Further exploration by other researchers regarding the above questions is needed to bridge the gaps in literature.

## Conclusion

The study found the mobile phone-based IVR system acceptable among caregivers. Attitude towards it was also found to be positive. The system improved caregivers’ communication with health professionals and supported them in making decisions concerning their children’s health. It also helped caregivers to learn about certain self-management healthcare practices of childhood diseases. During outbreaks of infectious diseases such as cholera, malaria, Ebola, Covid-19, etc., the mobile phone-based IVR system could serve as a surveillance tool to assist health authorities to act promptly to affected communities.

The high mobile phone access could also offer governments in sub-Saharan African countries the opportunity to utilize this innovative tool (IVR system) to harness the numerous challenges confronting their healthcare systems. However, proactive measures would need to be implemented to improve mHealth infrastructure; stable mobile cellular network, and reliable power supply,s which have served as the main barriers to mHealth system implementations in this region.
